# Lenalidomide Induces Immunomodulation in Chronic Lymphocytic Leukemia and Enhances Antitumor Immune Responses Mediated by NK and CD4 T Cells

**DOI:** 10.1155/2014/265840

**Published:** 2014-09-17

**Authors:** Andrea Acebes-Huerta, Leticia Huergo-Zapico, Ana Pilar Gonzalez-Rodriguez, Azahara Fernandez-Guizan, Angel R. Payer, Alejandro López-Soto, Segundo Gonzalez

**Affiliations:** ^1^Department of Functional Biology, IUOPA, University of Oviedo, Facultad de Medicina, Julian Claveria sn, 33006 Oviedo, Spain; ^2^Department of Hematology, Hospital Universitario Central de Asturias, C/Celestino Villamil s/n, 33006 Oviedo, Spain

## Abstract

Lenalidomide is an immunomodulatory drug with therapeutic activity in chronic lymphocytic leukemia (CLL). However, it has pleiotropic effects, and the mechanism of action responsible for its therapeutic activity has not been well defined yet. Herein, we show that lenalidomide treatment does not have an effect on the proliferation of leukemia cells, but it increases the proliferation of B cells from healthy donors. Lenalidomide did not exert a direct effect on the apoptosis of leukemia cells obtained from CLL patients, although it indirectly induced their apoptosis through the activation of nonmalignant immune cells. Thus, lenalidomide markedly increased the proliferation of NK and CD4 T cells. The effect of lenalidomide on NK cells was secondary to the induction of IL-2 production by CD4 T cells. Accordingly, depletion of T cells or blockade of IL-2 activity completely abrogated the proliferation of NK cells. Additionally, lenalidomide enhanced NK and NKT-like cell-mediated natural cytotoxicity against leukemia cells from CLL patients. Lenalidomide also upregulated CD20 expression on leukemia cells and, accordingly, it had a synergistic effect with rituximab on promoting antibody-dependent cell-mediated cytotoxicity against primary leukemia cells. Overall, these observations provide a support for combining lenalidomide with rituximab as a treatment in CLL.

## 1. Introduction

Chronic lymphocytic leukemia (CLL) is a heterogeneous disease, with a clinical presentation ranging from indolent to advanced stage disease. A therapeutic intervention is scarcely required in patients with indolent disease, whereas chemotherapy treatment is frequently required in patients with advanced stage disease. However, CLL is generally considered as an incurable disease and, consequently, the development of new therapeutic strategies is a key goal in this malignancy [1].

Increasing evidence demonstrates that the tumor microenvironment plays a critical role in CLL progression and therapy efficiency. The immune system is able to prevent cancer development, either by eliminating cancer cells prior to tumors becoming clinically detectable or by attenuating tumor progression [2, 3]. NK and T cells may mediate antitumor responses, particularly in the initial stages of the disease, which may affect disease progression [4, 5]. However, advanced disease patients develop multiple immune defects, including hypogammaglobulinemia, deregulation of the cytokine network, or impairment of T and NK cells function [6]. Nevertheless, targeting the immune system may represent a promising therapeutic strategy in CLL. Thus, chemotherapy is often combined with an anti-CD20 monoclonal antibody (rituximab) in patients with advanced stage disease, resulting in enhanced complete and overall response rates. The relevant mechanism of action of rituximab is the activation of NK cell-dependent antibody-dependent cell-mediated cytotoxicity (ADCC) against leukemia cells [7, 8].

Lenalidomide (Revlimid; Celgene) is an immunomodulatory drug that has shown a clinical effect in several hematological disorders including myeloma [9], myelodysplastic syndrome (MDS) [10], and CLL [11–14]. Lenalidomide displays a number of pharmacodynamic effects, but the main mechanism of action is not completely known and may vary depending on the disease. In multiple myeloma, lenalidomide exerts a direct cytotoxic effect on neoplastic plasma cells, inhibits cell adhesion, and induces changes in the bone marrow microenvironment [15]. In del(5q)MDS, lenalidomide directly affects erythroid progenitors [16]. In CLL, significant clinical responses, including molecular complete remissions in heavily pretreated patients, have been observed [12, 14]. It is noteworthy that lenalidomide does not directly induce the apoptosis of leukemic cells [17], but it regulates critical prosurvival and angiogenic cytokines (including IL-2, PDGF, and VEGF). Lenalidomide also stimulates antigen presentation, proliferation, and effector activity of T cells [18, 19] and may activate a minor cytotoxic population of T cells known as invariant or CD1d-restricted NKT cells [20, 21]. Furthermore, CLL cells incubated with healthy T cells inhibit immune synapse formation, where it is restored by lenalidomide treatment [22]. Additionally, lenalidomide increases NK cell proliferation, which correlates with clinical response [11, 23, 24] and augments NK cell-mediated ADCC against tumor cells [25, 26]. Likewise, clinical responses in CLL patients treated with lenalidomide correlated with a tumor flare reaction [18], which appears to be characteristic of this disease and may reflect a clinical manifestation of the enhancement of the immunogenic potential of tumors [14, 27].

The efficacy of lenalidomide in different malignant conditions may be explained by the existence of multiple mechanisms of action, different immune status, and specific pathogenesis of the disease. Unraveling the relevant mechanism of action is essential to optimize the treatment of patients and to develop new therapeutic strategies. Thus, in this study, we analyzed the mechanism of action underpinning the therapeutic activity of lenalidomide in CLL.

## 2. Material and Methods

### 2.1. Cell Isolation and Reagents

CLL patients (*n* = 17) fulfilling the diagnostic criteria for CLL [28] and healthy donors (*n* = 10) were analyzed in this study. These patients either were untreated or did not receive cytoreductive treatment within 6 months of the investigation. This study was approved by the ethics committee of our institution and informed consent was obtained from all patients and healthy donors.

Peripheral blood mononuclear cells (PBMCs) were purified by Ficoll gradient centrifugation from freshly isolated blood obtained from patients and donors. B cells were further purified using EasySep Human B Cell Enrichment Kit without CD43 Depletion (Stemcell Technologies) and NK cells were isolated from PBMCs by using the EasySep NK Cell Enrichment kit (Stemcell Technologies). The purity of B and NK cells (~90 to 95%) was assessed by flow cytometry.

PBMCs or purified immune cells were cultured in complete medium RPMI-1640 supplemented with 10% human AB serum, 2 mM L-glutamine, 100 U/mL penicillin, and 100 *μ*g/mL streptomycin (Sigma, St. Louis, MO) at 37°C in 5% CO_2_. Lenalidomide was obtained from Celgene and was dissolved in dimethyl sulfoxide (DMSO), and fresh lenalidomide was replaced every 72 hours in cell cultures. In some experiments, cells were treated with recombinant human IL-2 (rhIL-2) (Peprotech), anti-human IL-2 receptor (IL-2 sR*α*) blocking antibody (R&D systems), or cyclosporine A (CsA) (Sigma, St. Louis, MO). In all experiments incubation with DMSO was used as a control.

### 2.2. Flow Cytometry

Diagnosis of CLL was confirmed for each patient by flow cytometry, revealing a typical CD19^+^, CD20^+^, CD5^+^, CD23^+^, and Ig light chain (*κ* or *λ*) restricted phenotype of leukemia cells (Becton Dickinson). To determine immune cells subsets, cells were stained with anti-CD3-FITC, anti-CD4-PerCP, anti-CD8-CFBlue, anti-CD56-APC, and anti-CD20-PE (all from Immunostep) and anti-CD3-PECy7 (eBioscience) and isotype-matched control conjugates. The populations of immune cells were defined as follows: CD4 T cells were defined as CD3^+^CD4^+^ and CD8 T cells as CD3^+^CD8^+^; NK cells were identified as CD3^−^CD56^+^, NKT-like cells as CD3^+^CD8^+^CD56^+^, and leukemia cells as CD5^+^/CD19^+^. CD20 expression was quantified on CD5^+^/CD19^+^ cells.

To study the intracellular IL-2 production, PBMCs were incubated in complete culture media with GolgiStop (BD Biosciences) and with monensin (a protein transport inhibitor), for 4 hours. After that, cells were stained for surface antigens and, then, incubated with a fixation/permeabilization solution (BD Biosciences) prior to incubation with an anti-human IL-2-PerCP-Cy5.5 antibody (BD Biosciences).

Cells undergoing apoptosis were quantified by staining with annexin V-FITC according to the manufacturer's protocol (Immunostep). Cells were analyzed in a BD FACS Canto II cytometer and data were acquired and analyzed by using the FACS Diva Software.

### 2.3. Cell Proliferation Assay

Freshly isolated PBMCs from healthy donors and CLL patients were labeled with 5,6-carboxyfluorescein diacetate succinimidyl ester (CFSE) (Sigma, St. Louis, MO) at 1 *μ*M for 10 min at 37°C. Labeling was stopped with 5 volumes of complete media containing 10% fetal bovine serum (FBS). After 2 washes, cells were cultured at 2 × 10^6^ cells/mL in complete media containing 10% of human AB serum, and 1 *μ*M lenalidomide was added every 72 hours. After 3, 6, 9, 12, and 14 days of culture, cells were stained for CD19, CD3, CD4, CD8, and CD56 expression. Cell division was analyzed based on the decrease in CFSE staining, resulting from the dilution of the dye with each cell division.

### 2.4. NK Cell Cytotoxic Assays

CD107a lysosome-associated membrane protein-1 (LAMP-1) was used to measure NK-, NKT-like-, and CD8-cell cytotoxic activity. PBMCs from healthy donors or CLL patients stimulated with 1 *μ*M lenalidomide for 14 days were incubated with target cells at an effector : target (E : T) ratio of 5 : 1 in complete media supplemented with human AB serum and BD GolgiStop (BD Biosciences). As a positive control of degranulation, PBMCs were stimulated with PMA (50 ng/mL) and ionomycin (1 *μ*g/mL) (both from Sigma, St. Louis, MO). The anti-CD107a-PE antibody (BD Biosciences) was added to the plate during the incubation. Some experiments were made in the presence or absence of rituximab (20 *μ*g/mL). After the incubation, samples were stained for CD3, CD4, CD8, and CD56 expression and analyzed by flow cytometry.

### 2.5. Statistical Analysis

Continuous variables were compared with Mann-Whitney* U* test. Correlations between continuous variables were analyzed by Spearman correlation test. The *P* values *P* < 0.05 were considered statistically significant.

## 3. Results

### 3.1. Effect of Lenalidomide on Proliferation and Apoptosis of Leukemia Cells

**Figure 1:**
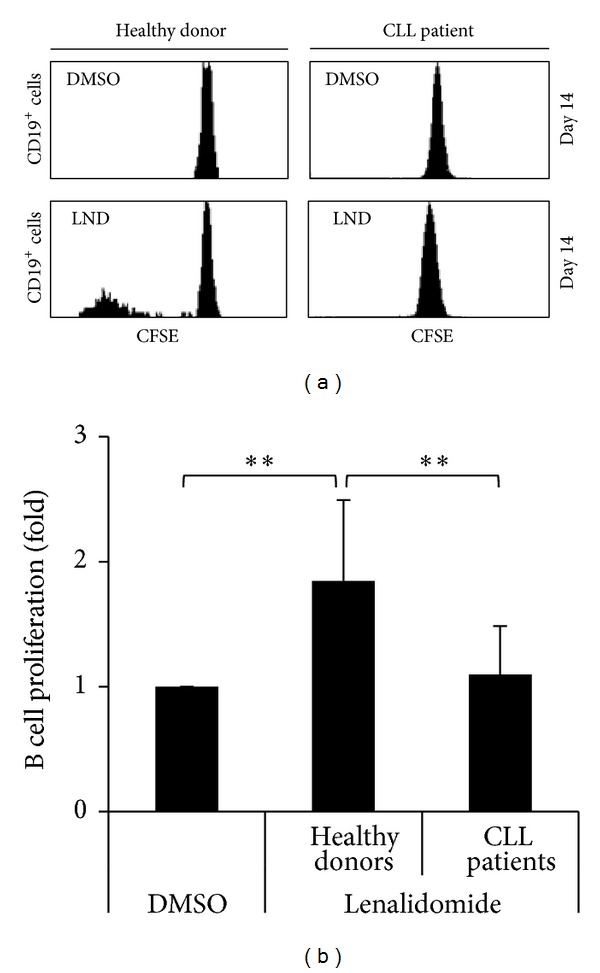
Effect of lenalidomide on the proliferation of B cells. (a) PBMCs obtained from healthy donors (*n* = 10) and CLL patients (*n* = 17) were labeled with CFSE and cultured with 1 *μ*M lenalidomide or DMSO for 14 days. CFSE fluorescence in B cells (gated as CD5^+^/CD19^+^) was analyzed by flow cytometry. The histograms show a representative CLL patient and a healthy donor. (b) The figure shows the compilation of the results obtained from healthy donors and CLL patients. Results are expressed as the fold induction of the percentage of proliferating B cells in lenalidomide-treated cells relative to the vehicle-treated control (**P < 0.01, Mann-Whitney* U* test).

The effect of lenalidomide on the proliferation of B cells was initially analyzed. PBMCs from healthy donors and CLL patients were cultured in presence of 1 *μ*M lenalidomide for 14 days and the proliferation of B cells was assessed by CFSE assay. As shown in Figures 1(a) and 1(b), lenalidomide did not affect the proliferation of leukemia cells, but the proliferation of B cells from healthy donors was significantly increased in the presence of this drug.

**Figure 2:**
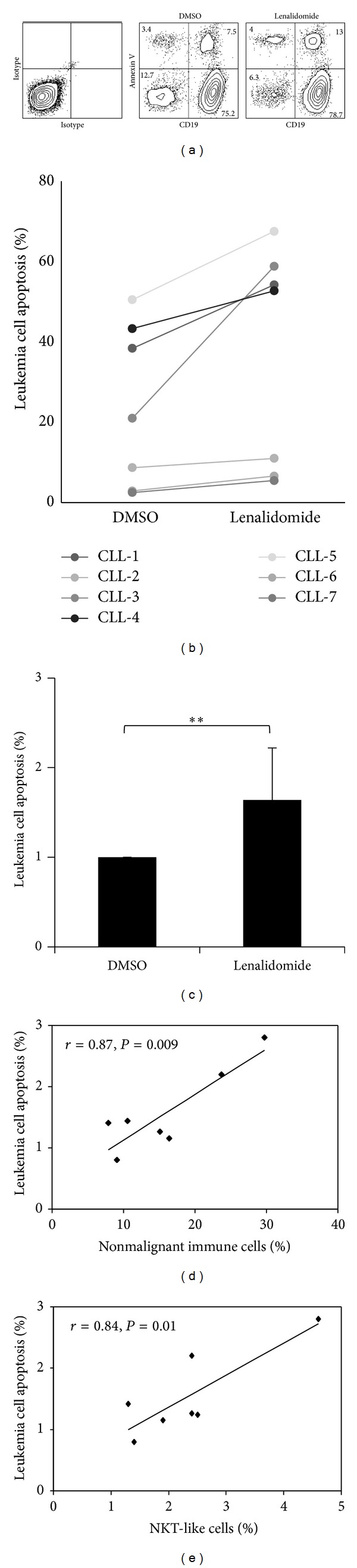
Effect of lenalidomide on the apoptosis of leukemia cells. (a) PBMCs from CLL patients (*n* = 7) were cultured in the presence of 1 *μ*M lenalidomide or DMSO for 7 days and apoptosis was analyzed by staining with annexin V. The figure shows the flow cytometric profile of annexin V staining of leukemia cells from a representative patient after lenalidomide treatment (numbers in the dot plot represent the percentage of cells). (b) PBMCs (ranging from 70% to 95% of leukemia cells) from CLL patients (*n* = 7) were cultured as detailed in *a* and apoptosis of leukemia cells was studied by annexin V staining. The scatterplot represents the percentage of apoptosis in DMSO versus lenalidomide-treated cells. (c) The bars represent the mean and the standard deviation of the fold induction of annexin V-positive leukemia cells (**P < 0.01, Mann-Whitney* U* test). (d) Correlation between annexin V staining of leukemia cells and the percentage of nonleukemia immune cells of CLL patients. (e) Correlation between annexin V labeling of leukemia cells and the percentage of NKT-like cells (CD3^+^CD8^+^CD56^+^) of CLL patients.

Next, we studied the effect of lenalidomide on the apoptosis of leukemia cells. PBMCs from CLL patients containing variable amounts of leukemia cells (ranging from 70% to 95%) and nonmalignant immune cells were used. No significant increase of apoptosis of leukemia cells from CLL patients was observed after 48 hours of treatment with 1 *μ*M lenalidomide (not shown). Nevertheless, after 7 days of treatment a significant effect of lenalidomide on the apoptosis of leukemia cells was detected (Figures 2(a), 2(b), and 2(c)). It is of note that the level of apoptosis on leukemia cells significantly correlated with the percentage of nonmalignant immune cells at day zero in CLL patients (*r* = 0.87, *P* = 0.009) (Figure 2(d)). Specifically, a strong correlation between apoptosis of leukemia cells and the percentage of NKT-like cells (CD3^+^CD8^+^CD56^+^) was observed (*r* = 0.84, *P* = 0.01) (Figure 2(e)).

### 3.2. Lenalidomide Enhances the Proliferation of NK Cells

**Figure 3:**
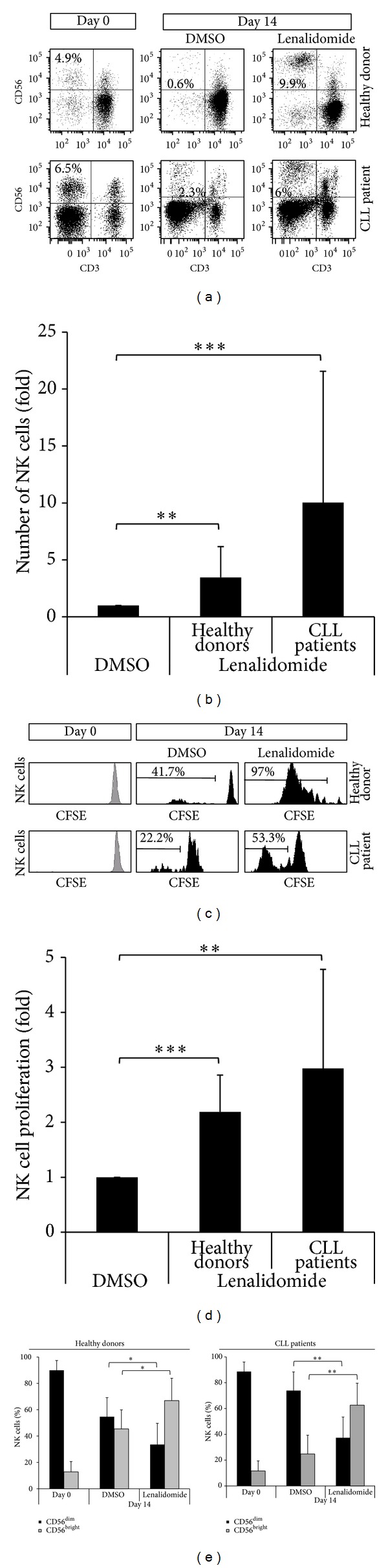
Effect of lenalidomide on NK cell proliferation. (a) Flow cytometric profile of NK cells (gated as CD3^−^CD56^+^) before and after stimulation with 1**** *μ*M lenalidomide for 14 days. One representative CLL patient and one healthy donor are shown. The numbers represent the percentage of NK cells. (b) The figure shows the compilation of the results obtained from CLL patients (*n* = 17) and donors (*n* = 10). Results are expressed as the fold induction of the percentage of NK cells of lenalidomide-treated cells relative to the vehicle-treated control (**P < 0.01; ***P < 0.001, Mann-Whitney* U* test). (c) PBMCs labeled with CFSE were cultured with 1**** *μ*M lenalidomide or DMSO for 14 days and CFSE expression was examined in NK cells (CD3^−^CD56^+^) by flow cytometry. One representative CLL patient and one healthy donor are shown. (d) The figure shows the compilation of the results obtained from CLL patients (*n* = 17) and healthy donors (*n* = 10). Results are expressed as the fold induction of the percentage of proliferative NK cells of lenalidomide-treated cells relative to the vehicle-treated control (**P < 0.01; ***P < 0.001, Mann-Whitney* U* test). (e) The figure shows the percentage of the two main subsets of NK cells (CD56^dim⁡^ and CD56^brigth^) after the treatment with 1 *μ*M lenalidomide or DMSO for 14 days. (*P < 0.05; **P < 0.01, Mann-Whitney* U* test).

In addition to the effect observed on B cells, it is noticeable that the percentage of NK cells significantly increased after 14 days of culture of PBMCs in the presence of lenalidomide (Figures 3(a) and 3(b)). Moreover, such increase of NK cells was higher in patients than in healthy donors (9.8- versus 3.4-fold induction). To analyze whether the increase of NK cells was due to cell proliferation, PBMCs from patients and healthy donors were CFSE-stained and cultured in the presence of lenalidomide for 14 days, and the proliferation of NK cells was examined by flow cytometry at days 3, 6, 9, 12, and 14. Remarkably, no effect was observed on the proliferation of NK cells before 12 days of treatment (not shown). Nevertheless, after 14 days, lenalidomide significantly increased the proliferation of NK cells from both healthy donors and CLL patients (Figures 3(c) and 3(d)), although the level of the induction was higher in patients than in healthy donors (2.98- versus 2.19-fold induction). In addition, there was a significant interpatient variation in the response to lenalidomide (ranging from no response to a 9.2-fold induction).

Interestingly, the main subsets of NK cells (CD56^bright^ and CD56^dim⁡^) proliferated in the presence of lenalidomide (not shown), although CD56^bright^ NK cells were more potently induced by lenalidomide than CD56^dim⁡^ NK cells (Figure 3(e)). Specifically, lenalidomide induced an inversion of the CD56^bright^/ CD56^dim⁡^ ratio from 0.82 to 1.99 in healthy donors and from 0.33 to 1.67 in CLL patients.

### 3.3. Lenalidomide Stimulates the Proliferation of T Cells

**Figure 4:**
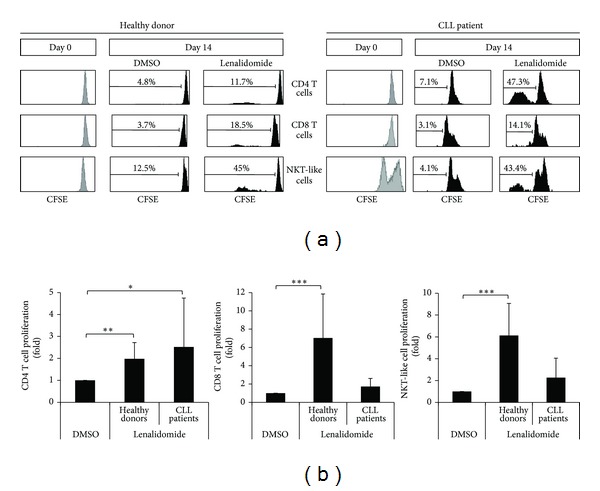
Effect of lenalidomide on the proliferation of T cell subsets. (a) The histograms show the CFSE expression of CD4 T cells, CD8 T cells, and NKT-like cells before and after stimulation with lenalidomide. PBMCs were labeled with CFSE and cultured with 1 *μ*M lenalidomide or DMSO for 14 days. CFSE expression in CD4 T cells (CD3^+^CD4^+^), CD8 T cells (CD3^+^CD8^+^), and NKT-like cells (CD3^+^CD8^+^CD56^+^) was examined by flow cytometry. One representative CLL patient and one donor are shown. (b) The figure shows the compilation of the results obtained from CLL patients (*n* = 17) and donors (*n* = 10). Results are expressed as the fold induction of the percentage of proliferative CD4 T cells, CD8 T cells, and NKT-like cells of lenalidomide-treated cells relative to the vehicle-treated control. (*P < 0.05; **P < 0.01; ***P < 0.001, Mann-Whitney* U* test).

We next examined the effect of lenalidomide on the proliferation of T cells (Figures 4(a) and 4(b)). It is of note that CD4 T cells were the most proliferative subset of T cells in CLL patients. The proliferation of CD4 T cell after 14 days of culture in the presence of lenalidomide was higher in CLL patients than in donors (2.52- versus 1.98-fold induction). Similar to NK cells, there was a marked interindividual variability in the proliferation of CD4 T cells; and a significant correlation between the percentage of CD4 T cells of CLL patients after lenalidomide treatment and the proliferation of NK cells was observed (*r* = 0.49, *P* = 0.04).

CD8 T cells also proliferated in response to lenalidomide, but the level of induction was higher in donors than in patients (7.03- versus 1.72-fold induction) (Figures 4(a) and 4(b)). Specifically, CD8 proliferated in 100% of healthy donors and 66% of CLL patients. Similar results were obtained with NKT-like cells, a subset of CD8 T cells (Figures 4(a) and 4(b)).

### 3.4. Induction of IL-2 Production by CD4 T Cells Is Required for the Enhancement of NK Cell and NKT-Like Cell Proliferation by Lenalidomide

**Figure 5:**
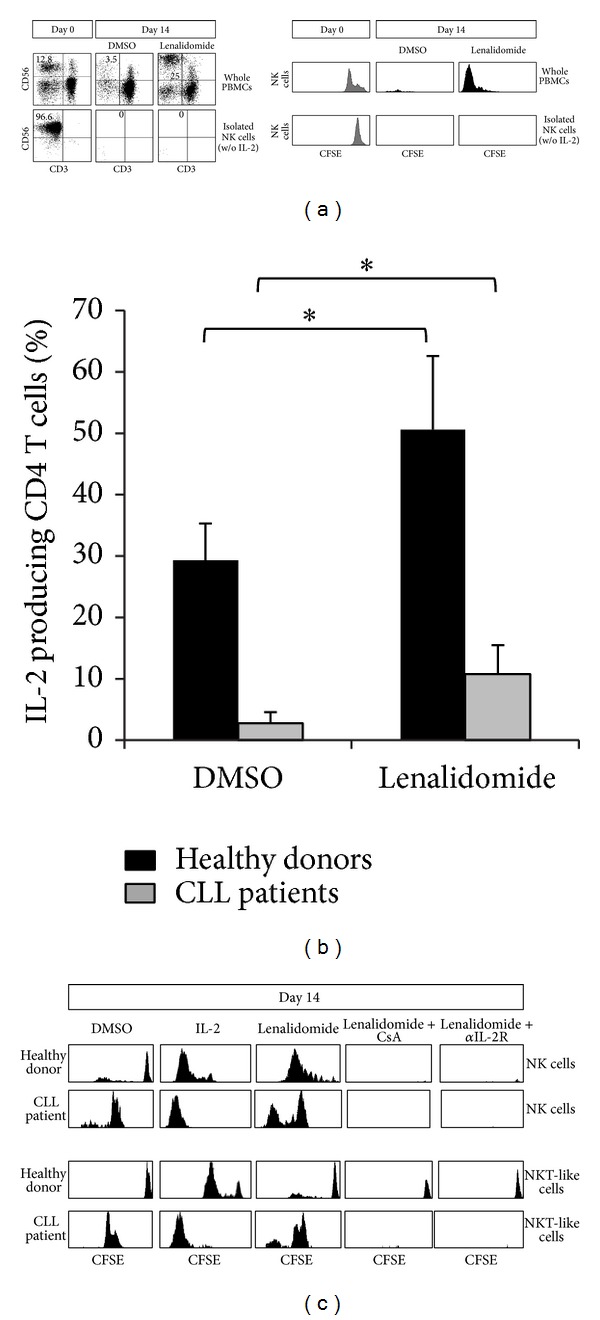
Production of IL-2 by CD4 T cells is required for lenalidomide-increased NK and NKT-like cells proliferation. (a) Whole PBMCs or purified NK cells (>95% of purity) from the same individual (*n* = 4) were labeled with CFSE and cultured with 1 *μ*M lenalidomide or DMSO for 14 days (in the absence of recombinant IL-2, w/o). The proliferation of NK cells (CD3^−^CD56^+^) was assessed by flow cytometry. The figure shows the cytometric profile of NK cells (gated as CD3^−^CD56^+^) before and after the stimulation with lenalidomide. The numbers represent the percentage of NK cells. The histograms represent the expression of CFSE in NK cells from one representative donor. (b) PBMCs from healthy donors (*n* = 4) and CLL patients (*n* = 4) were cultured in the presence of lenalidomide for 14 days and the intracellular production of IL-2 by CD4 T cells (CD3^+^CD4^+^) was analyzed by flow cytometry. The bars represent the mean and the standard deviation of the percentage of IL-2-producing CD4 T cells (*P < 0.05, Mann-Whitney* U* test). (c) PBMCs from healthy donors (*n* = 4) and CLL patients (*n* = 4) were stained with CFSE and cultured with DMSO, IL-2 (50 U/mL), or lenalidomide (1 *μ*M) in presence or absence of cyclosporine A (CsA) (1 *μ*M) or as anti-IL-2 blocking antibody (15 *μ*g/mL) for 14 days. PBMCs treated with IL-2 (50 U/mL) were used as a positive control of proliferation. Baseline peaks of CFSE are the same as in Figures 3(c) and 4(a). The expression of CFSE on NK cells and NKT-like cells (CD3^+^CD8^+^CD56^+^) was analyzed by flow cytometry. One representative healthy donor and one CLL patient are shown.

To unravel the mechanism of action underlying the induction of NK cell proliferation observed, we analyzed whether lenalidomide has a direct or an indirect effect on NK cell proliferation by comparing the effect of lenalidomide on whole PBMCs versus purified NK cells. The depletion of non-NK immune cells by negative selection completely abrogated the induction of NK cell proliferation in both CLL patients and donors (Figure 5(a)), suggesting that the effect of lenalidomide on NK cell proliferation was indirect. We next examined the effect of lenalidomide on the production of IL-2 by immune cells by intracellular staining and flow cytometry. As shown in Figure 5(b), lenalidomide treatment significantly induced the production of IL-2 by CD4 T cells. Furthermore, NK cell proliferation was abrogated in the presence of an anti-IL-2 receptor blocking antibody or cyclosporine A, indicating that the production of IL-2 was required for the proliferation of NK cells (Figure 5(c)). Similarly, IL-2 was also involved in the proliferation of NKT-like cells. Finally, we demonstrated that NK and NKT-like cell proliferation was induced by the treatment of PBMCs obtained from both patients and donors with IL-2 (Figure 5(c)). Overall, these results indicate that the production of IL-2 by CD4 T cells is required for the induction of the proliferation of NK and NKT-like cells by lenalidomide. It is worth mentioning that IL-2 was also able to induce the proliferation of NK cells obtained from CLL patients who did not respond to lenalidomide treatment.

### 3.5. Lenalidomide Increases NK Cell-Mediated Natural Cytotoxicity and ADCC against Primary Leukemia Cells

**Figure 6:**
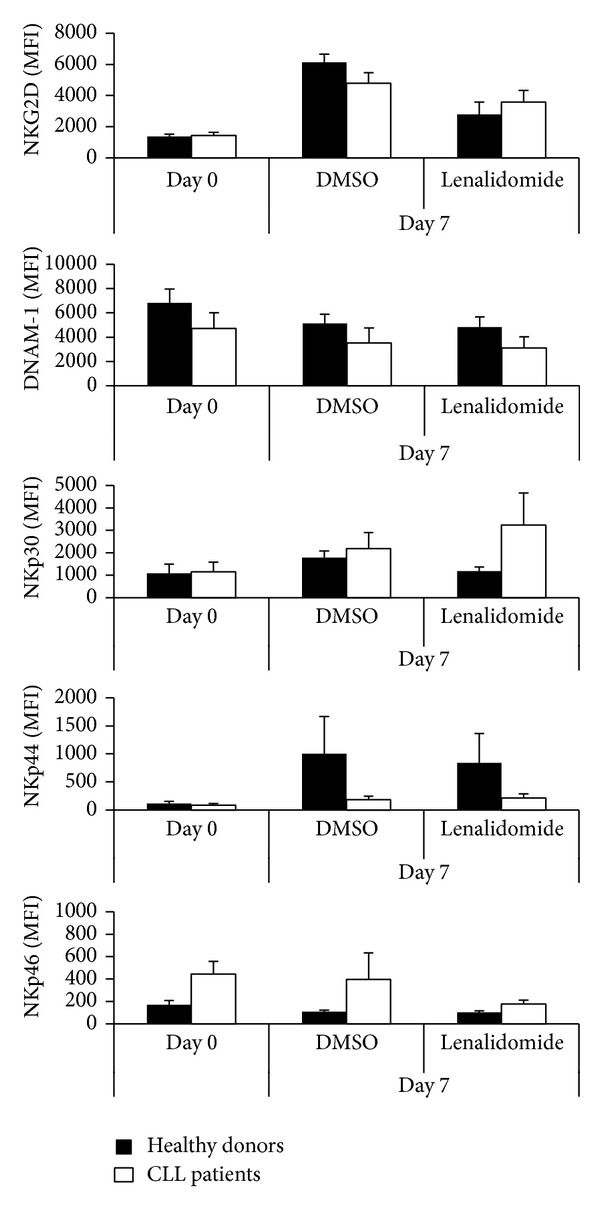
Effect of lenalidomide on the expression of NK cell activating receptors. PBMCs from healthy donors and CLL patients were treated with 1 *μ*M lenalidomide or DMSO for 7 days and the expression of NKG2D, DNAM-1, NKp30, NKp44, and NKp46 on NK cells (CD3^−^CD56^+^) was analyzed by flow cytometry. The figure shows the compilation of the results obtained from patients (*n* = 4) and donors (*n* = 3) before and after lenalidomide treatment. The bars represent the mean and standard deviation of the MFI.

First, the effect of lenalidomide on the expression of the main NK cell activating receptors was studied. To this end, PBMCs from patients and controls were incubated with lenalidomide for 7 days and the expression of NKG2D, DNAM1, NKp30, NKp44, and NKp46 was analyzed by flow cytometry. These experiments showed a significant increase in the expression of NKp30 on NK cells of CLL patients after lenalidomide treatment (Figure 6).

**Figure 7:**
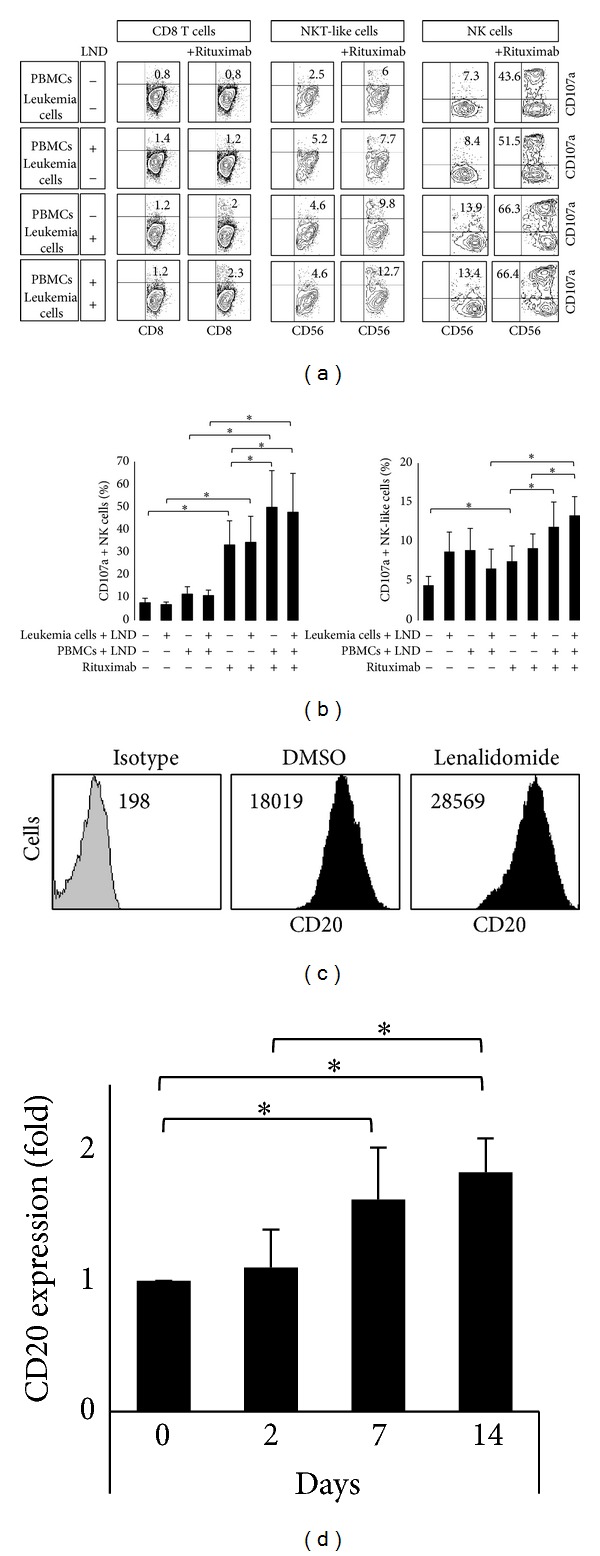
Lenalidomide enhanced natural cytotoxicity and ADCC against primary leukemia cells. (a) PBMCs from healthy donors (*n* = 4) and purified leukemia cells (>90% of purity) obtained from CLL patients (*n* = 4) were treated with 1 *μ*M lenalidomide or DMSO for 14 days. Lenalidomide and DMSO-stimulated PBMCs were cocultured with both lenalidomide and DMSO-stimulated leukemia cells at 5 : 1 E : T ratio in the presence or absence of rituximab (20 *μ*g/mL). The expression of CD107a was evaluated in NK cells (CD3^−^CD56^+^), CD8 T cells (CD3^+^CD8^+^), and NKT-like cells (CD3^+^CD8^+^CD56^+^) by flow cytometry. Representative dot plots showing CD107a expression on CD8, NKT-like, and NK cells of one representative experiment are shown. The numbers represent the percentage of CD107a^+^ cells for each subset analyzed. (b) The figure shows the compilation of the results obtained from patients and donors. The bars represent the mean and standard deviation of the percentage of CD107a^+^ NK cells and NKT-like cell (*P < 0.05; Mann-Whitney* U* test). (c) The histograms show the analysis of CD20 expression in leukemia cells after 14 days of lenalidomide treatment of a representative patient. Numbers in the histogram are the mean fluorescence intensity (MFI). (d) PBMCs from CLL patients (*n* = 4) were cultured with lenalidomide (1 *μ*M) for 2, 7, and 14 days and the expression of CD20 was analyzed on B cells (gated as CD5^+^/CD19^+^) by flow cytometry. Results are expressed as the fold induction of CD20 MFI in lenalidomide-treated cells relative to the vehicle-treated control (*P < 0.05; Mann-Whitney* U* test).

Next, we assessed whether lenalidomide modulates the cytotoxic activity of NK cells against purified leukemia cells. PBMCs from healthy donors incubated with lenalidomide or DMSO for 14 days were cocultured with purified leukemia cells obtained from CLL patients for 4 hours. Lenalidomide treatment significantly increased the cytotoxic activity of NK cells (1.5-fold induction) and NKT-like cells (2-fold induction) against primary leukemia cells (Figures 7(a) and 7(b)), but no effect was observed on CD8 T cells (shown in Figure 7(a)). Similar results were obtained using IL-2 or IL-15 treatment (not shown). The cytotoxic activity was not further increased when leukemia cells were also treated with lenalidomide, suggesting that the major effect of lenalidomide was exerted on the activity of immune cells.

The recognition of leukemia cells by NK cells may be increased with the use of antileukemic monoclonal antibodies [7, 8]. Thus, treatment of leukemia cells with rituximab (anti-CD20) in the absence of lenalidomide significantly increased the cytotoxic activity of NK (4.3-fold) and NKT-like cells (1.7-fold) against primary leukemia cells (Figures 7(a) and 7(b)). Moreover, lenalidomide significantly increased the rituximab-mediated cytotoxic activity of NK cells (1.5-fold) and NKT-like cells (1.6-fold) against leukemia cells (Figures 7(a) and 7(b)), but no effect was observed on CD8 T cells (shown in Figure 7(a)).

To characterize the underlying mechanism involved in the cooperative effect between lenalidomide and rituximab, we analyzed the effect of lenalidomide on CD20 expression on leukemia cells (CD5^+^/CD19^+^) after 2, 7, and 14 days of treatment. No clear effect on CD20 expression was observed after 48 hours of treatment, but it is noteworthy that a significant increase of CD20 expression was observed after 7 and 14 days of treatment (Figures 7(c) and 7(d)).

Our findings suggest that lenalidomide indirectly promotes the proliferation and cytotoxic activity of NK and NKT-like cells against primary leukemia cells. Additionally, lenalidomide induces ADCC to rituximab-treated leukemia cells of CLL patients. Overall, these data indicate that NK and NKT-like cells are relevant mediators of lenalidomide-driven apoptosis of leukemia cells in CLL.

## 4. Discussion

The activation of the antileukemic immune response represents a promising therapeutic option in CLL, particularly for relapsed patients. In this regard, lenalidomide is an immunomodulatory drug with significant therapeutic activity in CLL. However, lenalidomide has a pleiotropic activity and the relevant mechanism of action responsible for its therapeutic activity has not been well defined [11–14]. Our study indicates that the antileukemic activity of lenalidomide is not due to the direct cytotoxicity against leukemia cells, but rather it may imply indirect mechanisms through the activation of nonmalignant immune cells, particularly NK and CD4 T cells.

Our study shows a pleiotropic effect of lenalidomide on different types of immune cells including NK, T, and B cells. It is noticeable that, unlike normal B cells, transformed B cells do not proliferate in the presence of lenalidomide, suggesting that leukemia cells lose the capacity to respond to lenalidomide. Nevertheless our data suggest that NK and CD4 T cells may play a relevant role in the antileukemic effect of lenalidomide in CLL. Thus, lenalidomide was able to induce the proliferation of NK cells, and this effect was higher in CLL patients than in healthy donors. In agreement with our data, the NK cell number in patients was increased by lenalidomide treatment* in vivo* and pretreatment levels of NK cells correlated with the response to therapy in CLL [11, 23, 24]. Additionally, we observed that lenalidomide has an important effect on the proliferation of T cells, particularly CD4 T cells. The proliferation of CD4 T cells in response to lenalidomide treatment was also higher in patients than in healthy donors, and the percentage of CD4 T cells of CLL patients after lenalidomide treatment significantly correlated with the proliferation of NK cells. Additionally, the depletion of T cells abrogated the proliferation of NK cells suggesting a potential link between both cell types. It has been reported that lenalidomide facilitates the nuclear translocation of NFAT and AP-1, via activation of PI3K signaling, which results in IL-2 secretion by T cells [29]. Accordingly, our experiments show that the proliferation of NK and NKT-like cells is mediated by the production of IL-2 by CD4 T cells in CLL. Thus, the blockade of IL-2 activity completely abrogated the proliferation of NK and NKT-like cells. However, a remarkable observation in our study is the existence of a significant interindividual variation in the NK cell response among patients. It is worth mentioning that, in those CLL patients who did not respond to lenalidomide, IL-2 was able to induce the proliferation and the cytotoxic activity of NK cells, suggesting that the level of induction of IL-2 production by CD4 T cells by lenalidomide may be involved in this marked variation of response. The analysis of the therapeutic consequences of this variation and the potential predictive value of the* in vitro* analysis of NK cell proliferation deserves further investigation.

We observed that lenalidomide enhanced NK cell-mediated natural cytotoxicity against leukemia cells. This effect was mainly due to the activation of immune cells, since no further effect was achieved when leukemia cells were also treated with lenalidomide. Lenalidomide treatment increased the expression of NKp30 on NK cells from CLL patients, suggesting a role of this receptor in the increase of NK cell cytotoxicity observed in response to lenalidomide. It is also remarkable, but not unexpected, that lenalidomide had only a modest effect on promoting the antileukemic activity of NK cells against primary leukemia cells. In fact, leukemia cells express low levels of ligands of NK cell activating receptors, probably due to immune evasion mechanisms, being highly resistant to NK cell-mediated lysis [1, 30]. To increase the cytotoxic activity against leukemia cells it is necessary to favor the recognition of leukemia cells by NK and NKT-like cells. In line with this idea, lenalidomide is an attractive agent for combination with rituximab [25]. Our experiments showed a synergistic effect of lenalidomide with rituximab in promoting ADCC against leukemia cells, and this effect is supported by the fact that lenalidomide upregulated CD20 expression on leukemia cells. Overall, our findings provide a support for the combined use of lenalidomide with rituximab in the treatment of CLL patients and suggest that other treatments that increase the immunogenicity of tumor cells, for instance, by inducing the expression of ligands of NK cell receptors on leukemia cells such as histone deacetylase inhibitors [30], may be an attractive therapeutic strategy to be combined with lenalidomide. This clearly warrants further investigation.

In conclusion, our study indicates that the activation of CD4 T and NK cells is a key process underlying the therapeutic effect of lenalidomide in CLL, thus providing a rational support for optimizing and improving the efficacy of lenalidomide treatment in CLL patients.
